# Identification of an active RNAi pathway in *Candida albicans*

**DOI:** 10.1073/pnas.2315926121

**Published:** 2024-04-16

**Authors:** Elise Iracane, Cristina Arias-Sardá, Corinne Maufrais, Iuliana V. Ene, Christophe d’Enfert, Alessia Buscaino

**Affiliations:** ^a^Kent Fungal Group, School of Biosciences, Division of Natural Sciences, University of Kent, Canterbury CT2 7NZ, United Kingdom; ^b^Institut Pasteur, Université Paris Cité, Bioinformatic Hub, Paris F-75015, France; ^c^Institut Pasteur, Université Paris Cité, Fungal Heterogeneity Group, Paris F-75015, France; ^d^Institut Pasteur, Université Paris Cité, Institut national de recherche pour l’agriculture, l’alimentation et l’environnement USC2019, Fungal Biology and Pathogenicity Unit, Paris F-75015, France

**Keywords:** RNA interference, fungal pathogens, subtelomeres

## Abstract

RNA interference (RNAi) is a fundamental regulatory pathway with a wide range of functions, including regulation of gene expression and maintenance of genome stability. Although RNAi is widespread in the fungal kingdom, well-known species, such as the model yeast *Saccharomyces cerevisiae*, have lost the RNAi pathway. Until now evidence has been lacking for a fully functional RNAi pathway in *Candida albicans*, a human fungal pathogen considered critically important by the World Health Organization. Here, we demonstrated that the widely used *C. albicans* reference strain (SC5314) contains an inactivating missense mutation in the gene encoding for the central RNAi component Argonaute. In contrast, most other *C. albicans* isolates contain a canonical Argonaute protein predicted to be functional and RNAi-active. Indeed, using high-throughput small and long RNA sequencing combined with seamless CRISPR/Cas9-based gene editing, we demonstrate that an active *C. albicans* RNAi machinery represses expression of subtelomeric gene families. Thus, an intact and functional RNAi pathway exists in *C. albicans*, highlighting the importance of using multiple reference strains when studying this dangerous pathogen.

## Significance

Here, we present two significant findings that contribute to our understanding of *Candida albicans*, a life-threatening human fungal pathogen. First, we establish that the *C. albicans* reference strain is defective in RNA interference, a fundamental regulatory pathway. Second, we discover that, in contrast to the reference strain, the vast majority of *C. albicans* isolates contain an active RNA interference (RNAi) pathway that silences gene expression. Considering that RNAi plays central roles in reversibly governing gene expression, genome stability, drug resistance, and countering viral infections, our finding offers valuable insights into the biology of a dangerous fungal pathogen.

RNA interference (RNAi) is an ubiquitous set of related pathways in which small (~20 to 30 nucleotide; nt) noncoding RNAs (sRNA) orchestrate silencing of complementary nucleic acids using a diverse array of repressive mechanisms, such as assembly of transcriptionally silenced heterochromatin, RNA degradation, and translation inhibition (1, 2). RNAi can be triggered by double-stranded RNA (dsRNA) molecules from diverse sources including viral genomes, hairpin RNAs and overlapping sense and antisense transcripts. Two proteins are central for RNAi function: Dicer (Dcr) and Argonaute (Ago). Dcr is a ribonuclease III (RNaseIII) that processes dsRNA molecules into sRNA duplexes that are loaded into Ago proteins. The passenger strand of the duplex is selectively displaced and degraded, while the guide strand is retained, guiding Ago to complementary nucleic acids to target them for silencing.

Ago proteins are defined by the presence of Piwi/Argonaute/Zwille (PAZ) and P-element induced wimpy testes (PIWI) domains, often accompanied by less conserved N terminus and MID domains (3–5). The PAZ domain is essential for Ago activity as it binds sRNAs, driving targeting to complementary nucleic acids (6). The PIWI domain adopts an RNAse H-like fold and enables some, but not all, Ago proteins to cleave target RNAs complementary to the bound sRNA via a conserved catalytic DDED/H tetrad (5, 7–11).

Prokaryotic homologs of the key RNAi proteins have been identified and a fully functional RNAi machinery is present in the majority of eukaryotes, making it likely that RNAi arose in an early eukaryotic ancestor (12–14). Accordingly, RNAi is conserved throughout most of the fungal kingdom as an active RNAi machinery and is found in the main groups of fungi including the Ascomycota, Basidiomycota, and Zygomycota phyla (15). However, a few fungal species, including the model system *Saccharomyces cerevisiae*, the human fungal pathogen *Cryptococcus deuterogattii* and the plant pathogen *Ustilago maydis* have lost the RNAi pathway (16–20).

*Candida* species cause nearly half a million life-threatening infections annually and are the most common fungal pathogens in immunocompromized patients. Although over a dozen *Candida* species can be pathogenic, *Candida albicans* is the most prevalent (21, 22). Analysis of the RNAi pathway in *C. albicans* has been restricted to a single strain: the reference strain SC5314. This strain was isolated from a patient with systemic candidiasis and has been used as the standard laboratory strain for the *C. albicans* species worldwide since 1970 (23–25). Studies investigating RNAi in SC5314 have been inconclusive and suggest that *C. albicans* might not have a fully functional RNAi pathway. Indeed, although genome-wide mapping in the reference strain SC5314 has identified sRNA clusters that map to subtelomeric regions, it is unknown whether these sRNAs silence expression of complementary protein-coding or noncoding genes (26). Furthermore, expression of an artificial RNA hairpin, a common technique to trigger RNAi, failed to silence complementary nucleic acids in the SC5314-derived strain CAI4 (27).

The *C. albicans* SC5314 genome contains three genes encoding potential RNAi components: two Dicer proteins (Dcr1 and Cdl1) and one Argonaute protein (Ago1). Dcr1 and Cdl1 proteins belong to a family of noncanonical Dcr proteins found in several budding yeast species, including *Naumovozyma castellii* and *Vanderwaltozyma polyspora* (26, 28). These proteins are active RNAse III enzymes, structurally divergent from their canonical counterparts. They cleave dsRNA precursors into siRNAs using an “inside-out” mechanism (28, 29). *C. albicans DCR1* is an essential gene encoding a dual-function protein which acts as an Rnt1 enzyme, processing ribosomal RNA (rRNA), and snoRNA (29). Additionally, it also possesses canonical RNAi activity as it produces siRNAs in vitro and can mediate RNAi-silencing when expressed in *N. castellii* (29, 30). *CDL1* encodes an inactive RNAse III enzyme of unknown function (29). *C. albicans AGO1* encodes a nonessential protein whose function remains unknown (29).

Emerging evidence suggests that SC5314 may not always accurately represent the full spectrum of *C. albicans* biology. Indeed, recent studies have reported that *C. albicans* clinical isolates have diverse genomes and can be grouped in at least 17 genetic clusters (31–34). These isolates show significant variation in traits important for virulence and pathogenicity, including systemic infection levels in murine models, biofilm formation, cell wall remodeling, toxin secretion, and morphological plasticity (35–39).

In this study, we sought to establish the role of RNAi in *C. albicans* by analyzing a large number of *C. albicans* clinical isolates. We demonstrated that SC5314, the widely used reference strain, is unsuitable for studying RNAi as it contains the Ago1-K361 variant which represents an inactivating missense mutation. Importantly**,** unlike the reference strain, the vast majority of *C. albicans* clinical isolates are predicted to have a functional Ago1-E361 protein and thus be RNAi-active. By focusing on clinical isolates with active RNAi, we revealed a role for RNAi in *C. albicans,* demonstrating that it acts at subtelomeres to repress expression of the telomere-associated (*TLO*) gene family.

## Results

### The RNAi Machinery Is Not Associated with Gene Silencing in the *C. albicans* Reference Strain SC5314.

**Fig. 1.**
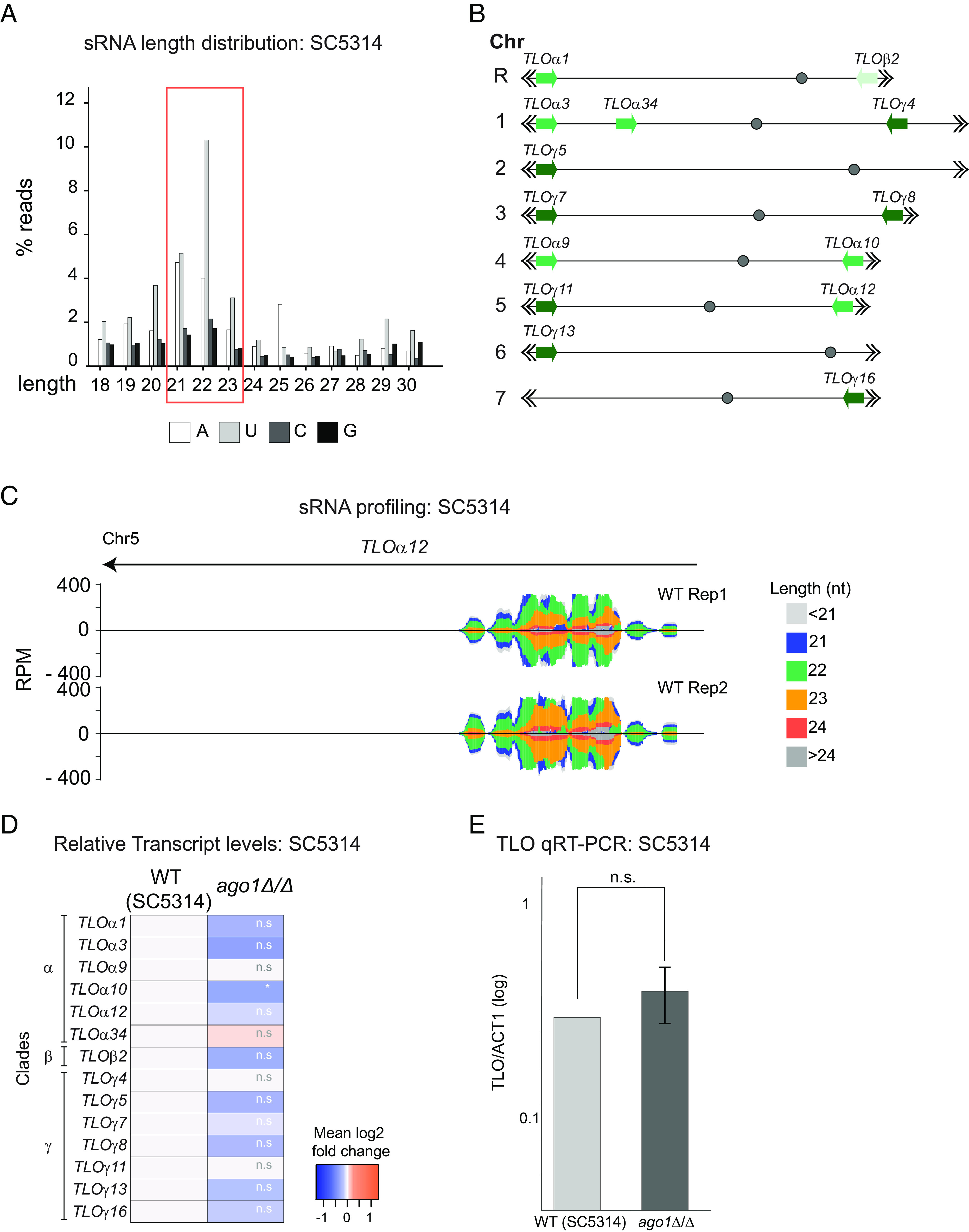
The RNAi machinery is not associated with gene silencing in the *C. albicans* reference strain SC5314. (*A*) Length distribution of sequencing reads representing small RNAs (18 to 30 nucleotides) with the indicated 5′ end nucleotide. (*B*) Graphical representation of *TLO* genes across *C. albicans* chromosomes. (*C*) Small RNA profiling of locus *TLOα12* in two biological replicates of WT (Rep1 and Rep2). (*D*) Heatmaps display relative transcript levels (log2 fold changes) from RNA-seq experiment in SC5314 *ago1*Δ/Δ compared to SC5314 WT for *TLO l*oci; n.s. = not significant; *P < 0.05 (*E*) Relative *TLO* transcript level from qRT-PCR in WT SC5314 and *ago1*Δ/Δ using pan-*TLO* primers. Graph bars: average of three biological replicates; error bars: SD; n.s. = not significant.

To assess the role of *C. albicans* RNAi, we investigated whether regions of the *C. albicans* genome that have sRNAs in wild-type cells are up-regulated in the absence of the key RNAi component Ago1. We sequenced sRNAs (sRNA-seq) isolated from the *C. albicans* SC5314 reference strain and mapped them to the *C. albicans* genome, confirming previous findings and identifying subtelomeric *TLO* genes as major sRNA (21 to 23 nt) hotspots (Fig. 1 *A*–*C* and *SI Appendix*, Fig. S1) (26). *TLO* genes are 14 closely related paralogues encoding Med2, a subunit of the Mediator transcriptional regulation complex (40). To investigate a potential link between siRNA clusters and RNAi-mediated gene silencing, we generated an *AGO1* deletion mutant (*ago1*Δ/Δ) by CRISPR-Cas9 genome editing. We focused on Ago1 because it is the key protein eliciting gene silencing and, unlike Dcr1, is not essential (29). Deleting *AGO1* in SC5314 does not affect the growth at 30 °C or 37 °C in rich Yeast-Extract Peptone Dextrose (YPD) media (*SI Appendix*, Fig. S2). Sequencing of long coding and noncoding RNAs (RNA-seq) in WT and *ago1*Δ/Δ revealed that deletion of *AGO1* does not lead to increased transcript levels of sRNA-rich loci (Fig. 1*D* and *SI Appendix*, Fig. S1). RT-qPCR analysis confirmed that deletion of *AGO1* does not lead to increased *TLO* mRNA levels (Fig. 1*E*). Furthermore, although we detected some differences in the gene expression profile of WT and *ago1*Δ/Δ strains, we did not detect sRNAs in WT cells for genes that were up-regulated in *ago1*Δ/Δ (Datasets S1 and S2). These results suggest that the RNAi machinery is not involved in transcriptional or posttranscriptional silencing in the *C. albicans* reference strain SC5314.

### An RNAi-Inactivating Point Mutation Is Present in the Laboratory Strain SC5314 but Not in the Vast Majority of *C. albicans* Clinical Isolates.

**Fig. 2.**
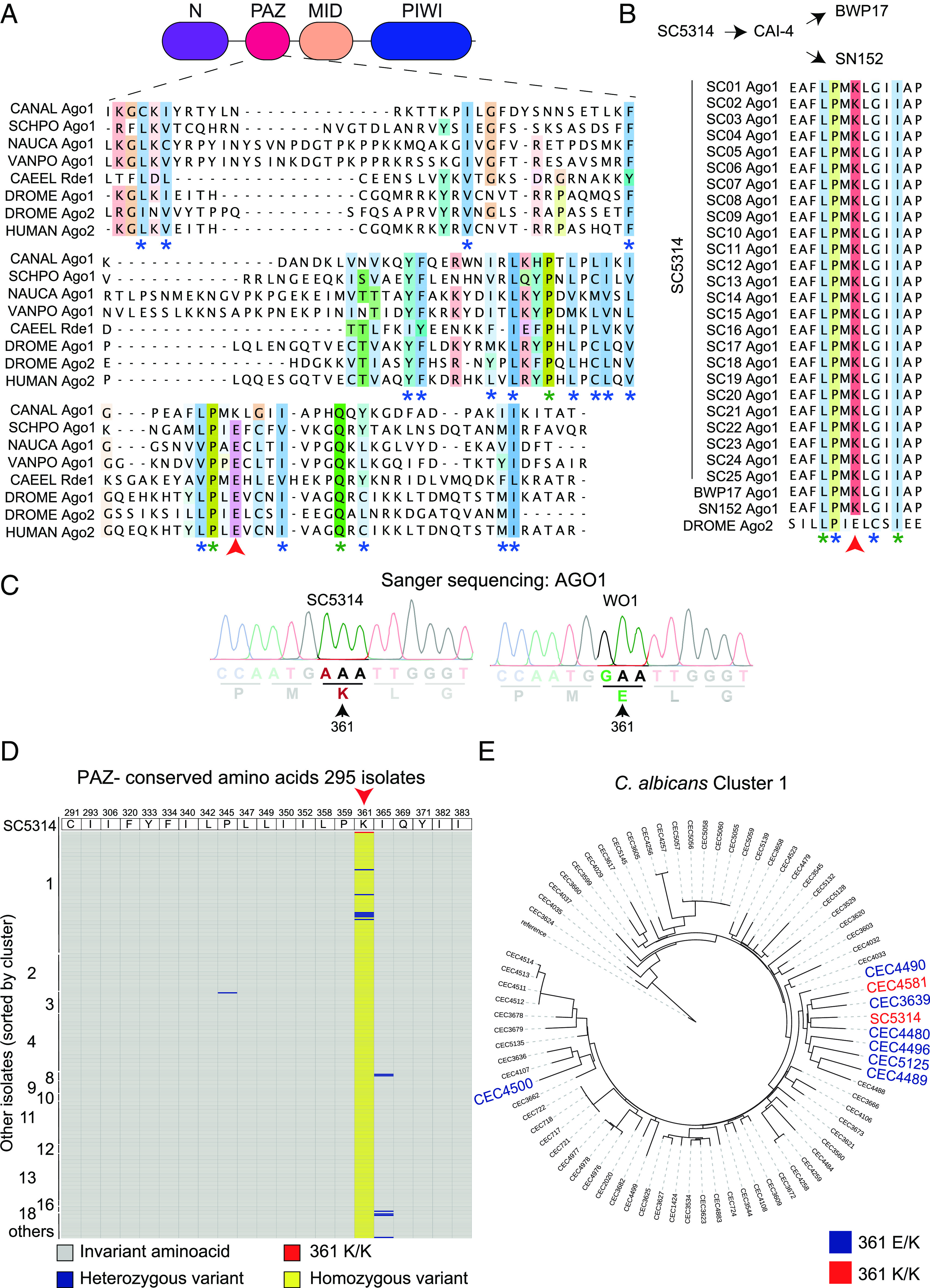
An RNAi inactivating point mutation is present in the laboratory strain SC5314 but not in the vast majority of *C. albicans* clinical isolates. (*A*) Schematic representation of CaAgo1 and alignment of PAZ domain from 8 eukaryotic species. CANAL: *C. albicans* SC5314 Ago1 aa 289-387; SCHPO: *Schizosaccharomyces pombe* Ago1 aa 240-349; NAUCA: *Naumovozyma castellii* Ago1 aa 600-722; VANPO: *Vanderwaltozyma polyspora* Ago1 aa 562-688; CAEEL: *Caenorhabditis elegans* Rde-1 aa 349-443; DROME: *Drosophila melanogaster* Ago1 aa 372-470 and Ago2 637-737; HUMAN: *Homo sapiens* Ago2 aa 263-370. Alignment visualized on Jalview; residues colored by functional conservation. Blue stars are conserved residues; green stars are identical residues; red arrow indicated K361 in *C. albicans* SC5314. (*B*) Alignment of Ago1 aa 355-367 of 25 *C. albicans* SC5314 isolates, BWP17, SN152 and *D. melanogaster* Ago2 aa 708-720 (DROME). Blue stars are conserved residues; green stars are identical residues; red arrow indicated K361 in *C. albicans* SC5314. (*C*) Chromatograms from Sanger sequencing results of SC5314 and WO-1 isolates focused on nucleotides 1,075 to 1,089. (*D*) Residues 291 to 383 of AGO1 PAZ domain from 295 *C. albicans* isolates compared to the SC5314 reference sequence (*Top* of the graph). The isolates are sorted by clusters. Red: 361 K/K; grey: invariant residues; blue: heterozygous variant; yellow: homozygous variant. Red arrow shows K361 from SC5314. (*E*) Phylogenetic tree of cluster 1 strains. In blue: heterozygous variants for Ago1-E/K 361; in red: homozygous variants for Ago1-K/K361. Tree constructed with RAxML using data for 215794 high-confidence SNPs available for the 295 *C. albicans* isolates and strain SC5314.

The observation that *C. albicans* Ago1 has no impact on gene silencing strongly implies that RNAi is inactive in this fungal pathogen. Given evidence that *C. albicans* Dcr1 is active (29), we focused on *C. albicans* Ago1 as the possible root cause of RNAi deficiency. Sequence alignment and AlphaFold structural prediction (41) indicate that *C. albicans* Ago1 possesses the canonical organization of Ago proteins with conserved PAZ, MID, and PIWI domains, along with a less conserved N domain (Fig. 2*A* and *SI Appendix*, Fig. S3). We conducted a comparative amino acid sequence alignment between *C. albicans* Ago1 (strain SC5314) and well-characterized Ago proteins across diverse species, focusing on the conserved PAZ, MID, and PIWI domains (Fig. 2*A* and *SI Appendix*, Figs. S4 and S5). The majority of functional amino acid residues conserved between species are present in *C. albicans* reference strain SC5314, with one important exception: the PAZ domain contains a lysine at position 361 (K361) which deviates from the universally conserved glutamate (E361) present in the PAZ domain of all other eukaryotic Ago proteins (Fig. 2*A*). This is a critical variation: Indeed, in *Caenorhabditis elegans,* the corresponding E-to-K change in the Argonaute protein Rde-1 abolishes RNAi activity and an *RDE-1^E-to-K^*mutation was isolated in one of the first genetic screens aimed at identifying RNAi factors (42). Furthermore, structural analysis of *Drosophila melanogaster* Ago2 demonstrated that this conserved E residue is required to stabilize the PAZ domain fold and is critical for Ago2 function (43). Comparative analysis of Ago1 protein sequences across 25 SC5314 strains sourced from 17 different laboratories and the American Type Culture Collection (44) indicated that all analyzed strains contain the putative RNAi inactivating mutation (G to A; Chr4 nt 1408146) in the *AGO1* gene, demonstrating that the Ago1-K361 variant did not arise recently but is an intrinsic feature of the standard laboratory SC5314 strain (Fig. 2*B* and Dataset S3). Likewise, Ago1 proteins encoded in genomes of SC5314-auxotrophic derivatives, BWP17 and SN152, contain the Ago1-K361 variant and are predicted to have an inactive RNAi pathway (Fig. 2*B* and Dataset S3).

To determine whether our finding that the reference *C. albicans* strain is likely to be RNAi defective accurately represents the entire species, we analyzed sequences from additional isolates. Sanger sequencing indicated that the second-most used *C. albicans* isolate, the mating competent WO-1 strain, does not contain the RNAi-inactivating variant Ago1-K361 (Fig. 2*C*). Surprisingly, a comparative analysis of Ago1 sequences across a large panel (n = 295) of *C. albicans* clinical isolates belonging to different *C. albicans* genetic clusters (45) (Fig. 2*D* and Dataset S4) revealed that the majority of these isolates (97%: 287/295 isolates) contain an RNAi-active Ago1-E361 variant. Only eight isolates (CEC3639; CEC5125; CEC4489; CEC4490; CEC4496; CEC4480; CEC4500; and CEC4581) contain the Ago1-K361 variant; seven of these strains (2.4%) are likely to be RNAi active, as they are heterozygous (Ago1-E361/Ago1-K361), while only one isolate (0.7%–CEC4581) is predicted to be RNAi inactive as it is homozygous for the Ago1-K361 variant similar to SC5314. Phylogenetic analyses strongly suggest that two events gave rise to these mutations: one event leading to CEC4500 and another leading to eight strains (six heterozygous and two homozygous) situated within the same branch of the phylogenetic tree (Fig. 2*E*). The sequence comparison analysis also revealed that the vast majority of analyzed *C. albicans* isolates do not contain any additional inactivating point mutations in the *AGO1* gene. Indeed, amino acids conserved across diverse species, including the catalytic DDEH/D tetrad essential for Ago1 slicing activity, are present in all strains. CEC4526 is the only exception as it contains a homozygous point mutation in a conserved amino acid of the PIWI domain (Fig. 2*D* and *SI Appendix*, Fig. S5). Our results show that of the 296 strains tested, only two strains, including the reference strain, appear to be RNAi inactive. Thus, until now, the role of RNAi in *C. albicans* biology has been overlooked due to the over-reliance of the SC5314 reference strain.

### RNAi Silences Expression of Subtelomeric Genes in *C. albicans* Clinical Isolates Not Related to the SC5314 Reference Laboratory Strain.

**Fig. 3.**
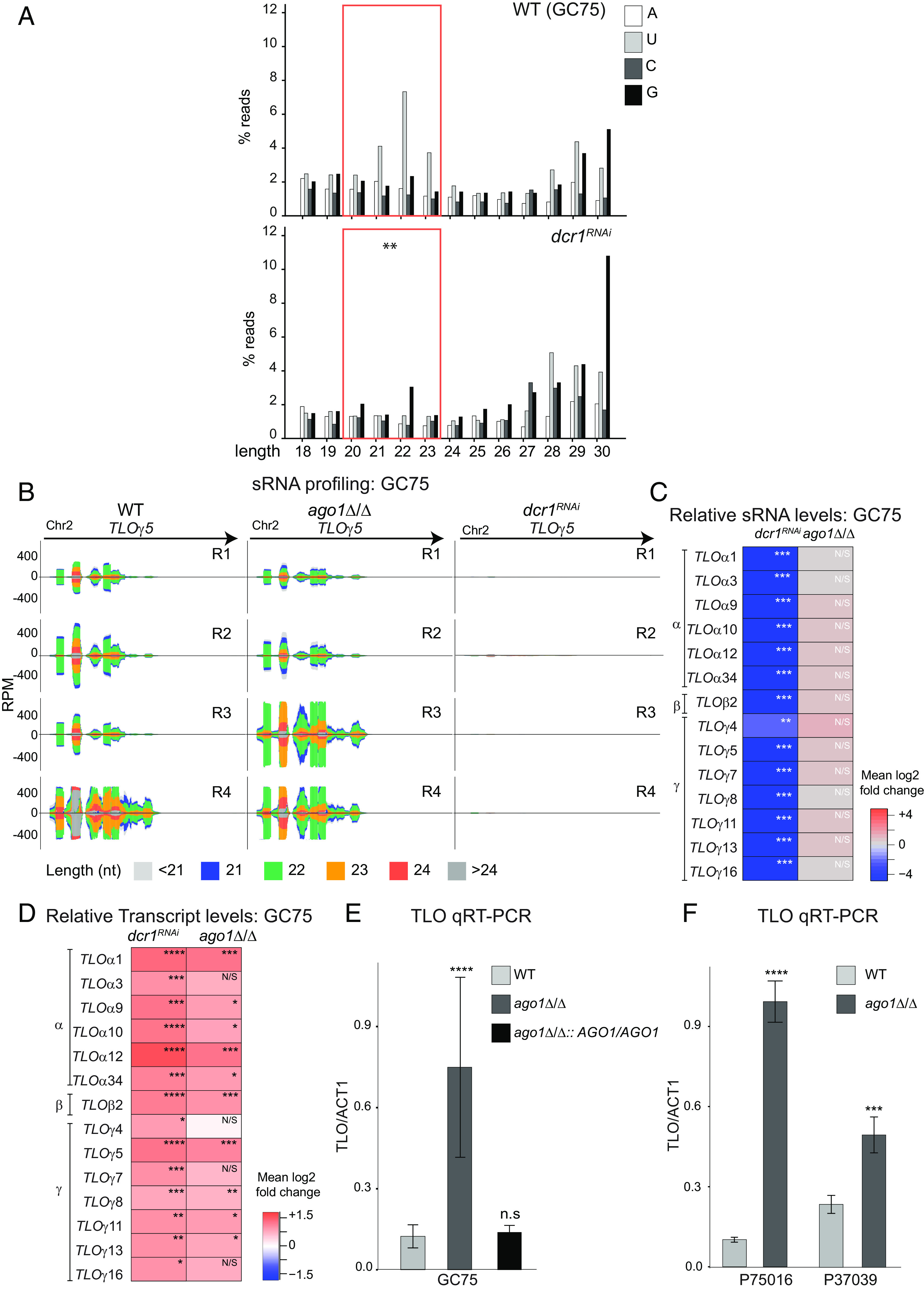
RNAi silences expression of subtelomeric genes in *C. albicans* clinical isolates not related to the reference laboratory SC5314 strain. (*A*) Length distribution of sequencing reads representing small RNAs (18 to 30 nt) with the indicated 5′ end nucleotide for WT (GC75) and *dcr1^RNAi^* (GC75). The total of 20-23 nt sRNA between samples is statistically different (*P* = 0.00244). (*B*) Small RNA profiling of locus *TLOγ5* in four biological replicates (R1, R2, R3 and R4) of WT (GC75), *ago1*Δ/Δ and *dcr1^RNAi^* strains. (*C*) Heat map of sRNA log2 fold change in *dcr1^RNAi^* (GC75) and *ago1*Δ/Δ (GC75) for at *TLO* loci from sRNA-seq data. n.s. = not significant; *P < 0.05, **P < 0.005, ***P < 0.0005, ****P < 0.00001. (*D*) Heatmaps display relative transcript levels (log2 fold changes) from RNA-seq experiment in GC75 *dcr1^RNAi^*and *ago1*Δ/Δ strains; n.s.= not significant; *P < 0.05, **P < 0.005, ***P < 0.0005, ****P < 0.00001. (*E*) Relative in GC75 WT, *ago1*Δ/Δ and *ago1*Δ/Δ: *AGO1/AGO1* strains using pan-TLO primers; Graph bars: average of three biological replicates; error bars: SD; ****P < 0.00001; n.s. = not significant. (*F*) Relative *TLO* transcript level from qRT-PCR in P75016 and P37039 WT strains and their corresponding *ago1*Δ/Δ strains using pan-*TLO* primers. Graph bars: average of three biological replicates; error bars: SD; n.s. = not significant; *P < 0.05, **P < 0.005, ***P < 0.0005, ****P < 0.00001.

In order to assess the impact of removing RNAi from *C. albicans,* we focused on a clinical isolate, GC75, (46–48) predicted to be RNAi active, undertaking deletion of genes encoding RNAi components Ago1 and Dcr1 by CRISPR-Cas9 genome editing. We generated an *AGO1* deletion mutant strain (*ago1*Δ/Δ), but could not obtain a *DCR1* deletion mutant, suggesting that, as in SC5314, *DCR1* is an essential gene in the clinical isolate GC75 due to its dual function as the Rnt1 ribonuclease and RNAi component (29). We therefore generated a RNAi-specific Dcr mutant (*dcr1^RNAi^*) by deleting the second dsRNA-Binding Domain which is required for in vivo RNAi activity of other noncanonical Dcr proteins but absent from Rnt1 proteins (28) (*SI Appendix*, Fig. S6 *A* and *B*). Although the *dcr1^RNAi^* strain is viable, it has a slower growth rate relative to the WT GC75 strain at both 30 °C and 37 °C (*SI Appendix*, Fig. S6*C*). Genome-wide sRNA sequencing supports the hypothesis that *dcr1^RNAi^* is RNAi defective. Indeed, sRNAs enriched in 21-23-mers starting with a 5′U, a bias typical of RNAi products, were prominent in the WT GC75 strain but dramatically reduced in the *dcr1^RNAi^*strain (Fig. 3*A*) (49). If RNAi silences specific genes, we expected that genes that are targeted by RNAi would have high levels of sRNAs in WT cells compared to *dcr1^RNAi^*mutant strains. These genes are predicted to have higher gene transcript levels in the *ago1*Δ/Δ and *dcr1^RNAi^*strains when compared to the WT GC75 isolate. Thus, we quantified levels of long coding and noncoding RNAs in the WT, *ago1*Δ/Δ and *dcr1^RNAi^*strains. In parallel, we quantified the levels of short RNAs that are detected in the same set of strains. We detected 133 coding and noncoding genes that have higher transcript levels and reduced sRNAs in *dcr1^RNAi^* compared to WT. In contrast, the deletion of *AGO1* results in 276 differentially expressed coding and noncoding genes when compared to the WT strain. Among these, 152 exhibit higher transcript levels, while 124 show lower transcript levels relative to WT (Dataset S1). To unequivocally determine the role of the RNAi machinery in gene silencing, we identified genes i) to which sRNAs map in WT but the levels of which are significantly reduced in the *dcr1^RNAi^*mutant and ii) are significantly up-regulated in the *ago1*Δ/Δ and *dcr1^RNAi^*mutants when compared to the WT GC75 strain. Only one set of genes met these criteria: the *TLO* gene family located at subtelomeric regions. Indeed, sRNA clusters mapping to *TLO* genes were abundant in the WT strain at levels similar to those in the reference strain SC5314 (Fig. 3*B* and *SI Appendix*, Fig. S6*E*). Importantly, these sRNA clusters were dramatically reduced in the *dcr1^RNAi^* strain, but not in the *ago1*Δ/Δ strain, as expected for Dicer-dependent sRNA formation (Fig. 3 *B* and *C* and Dataset S1) (50). Furthermore, *TLO* transcript levels were elevated in *ago1*Δ/Δ and *dcr1^RNAi^*strains compared to WT (Fig. 3 *D* and *E*). Genome-wide transcriptome data suggest that individual *TLO* genes respond differently to lack of RNAi, but it is difficult to establish whether specific *TLO* genes are preferential RNAi targets due to high sequence similarity of *TLO* genes coupled with short Illumina reads. Since transposable elements and centromeric regions are common RNAi targets in other organisms (51–53), we examined *C. albicans* transposons and centromeres for the presence of Dcr-dependent sRNA clusters. While we could not identify any sRNA clusters matching centromeric regions (<0.0007%), we did detect some Dcr-dependent sRNA clusters mapping to LTR and non-LTR retrotransposons, as well as DNA transposons (*SI Appendix*, Fig. S7). However, the sRNA peaks associated with transposons are much smaller than those matching the *TLO* genes. Additionally, we did not detect higher transcript levels originating from transposon elements in the absence of *AGO1* (*SI Appendix*, Fig. S7).

*TLO* overexpression in the absence of *AGO1* was confirmed by qRT-PCR analysis (Fig. 3*E*). Importantly, *TLO* transcript levels were reduced to WT levels when *AGO1* was reintroduced at its endogenous locus, establishing that the lack of the key RNAi component Ago1 leads to *TLO* overexpression (Fig. 3*E*). Finally, deletion of *AGO1* from two additional non-SC5314 clinical isolates (P75016 and P37039) led to elevated *TLO* transcript levels similar to those observed in the clinical isolate GC75 and in net contrast with those of the reference strain SC5314 (Figs. 1*E* and 3*F*).

Taken together these results demonstrate that, in contrast to the reference strain SC5314, RNAi is active in all three clinical isolates tested, and likely in others, where it acts to silence subtelomeric *TLO* genes.

### Ago1-K361 Variant Drives RNAi Deficiency in the Reference Strain SC5314.

**Fig. 4.**
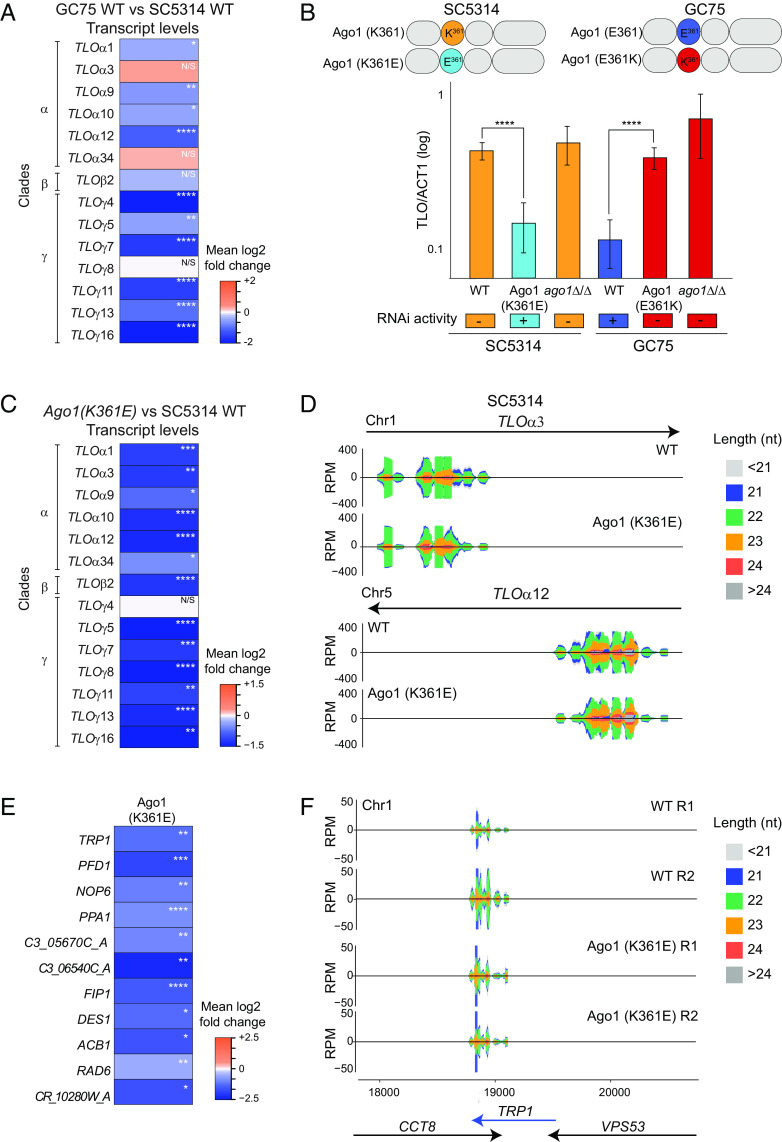
Ago1-K361 variant drives RNAi deficiency in the SC5314 laboratory reference strain. (*A*) Heatmap of relative *TLO* transcript levels (log2 fold changes) from RNA-seq experiment in GC75 WT compared to SC5314 WT (*B*) *Top*: Graphical representation of Ago1 variants in SC5314 and GC75; *Bottom*: qRT-PCR of TLO expression in SC5314 background [wild type, RNAi active strain (Ago1-K361E) and *ago1*Δ/Δ] and in GC75 background [wild type, RNAi inactive strain (Ago1-E361K) and *ago1*Δ/Δ]. Graph bars: average of three biological replicates; error bars: SD. (*C*) Heatmaps display relative transcript levels (log2 fold changes) from RNA-seq experiment in SC5314 Ago1-K361E compared to SC5314 WT. (*D*) Small RNA profiling of loci *TLOα3* and *TLOα12* in WT (SC5314) and Ago1-K361E (SC5314) strains. (*E*) Heatmap of relative transcript levels (log2 fold changes) from RNA-seq experiment in SC5314 Ago1-K361E compared to SC5314 WT. (*F*) Small RNA profiling of the TRP1 locus in two biological replicates (R1 and R2) of WT (SC5314) and Ago1-K361E (SC5314) strains. For all: n.s. = not significant; *P < 0.05, **P < 0.005, ***P < 0.0005, ****P < 0.00001.

Quantification of *TLO* transcript levels indicates they are significantly higher in SC5314, as expected in an RNAi mutant, compared to other non-SC5314 clinical isolates which have the canonical E amino acid at position 361 [Fig. 4*A* and *SI Appendix*, Fig. S8; (54)]. These data further support our hypothesis that the reference strain SC5314 is RNAi deficient because of an inactivating missense point mutation in *AGO1*.

To establish whether Ago1-K361 is responsible for the RNAi deficiency in *C. albicans*, we sought to restore RNAi in SC5314 by generating a strain encoding an Ago1-K361E variant (Ago1-K361E) at its endogenous locus. In parallel, we investigated whether the Ago1-K361 variant is sufficient to abolish RNAi activity in the clinical isolate GC75 by generating an Ago1-E361K strain (Fig. 4*B*). Quantification of *TLO* transcript levels by qRT-PCR analysis demonstrates that *TLO* transcript levels were significantly reduced in the engineered SC5314 Ago1-K361E strain relative to SC5314 and significantly higher in the GC75 Ago1-E361K strain relative to GC75 (Fig. 4*B*). Genome-wide quantification of long and sRNA levels confirm that the reintroduction of Ago1-K361E into SC5314 reduces *TLO* transcripts abundance without affecting sRNA levels (Fig. 4 *C* and *D* and *SI Appendix*, Fig. S9). A total of 464 coding and noncoding transcripts are significantly down-regulated in the Ago1-K361E strain. Among those, 11 genes are associated with sRNAs though at a variable and lower levels than *TLO-*associated sRNAs (Fig. 4 *E* and *F* and *SI Appendix*, Fig. S10).

In summary, these data demonstrate that the Ago1-K361 variant is responsible for the RNAi deficiency of the SC5314 reference strain in *C. albicans.*

## Discussion

In this study, we demonstrated that the *C. albicans* reference strain commonly used in laboratories worldwide is deficient in one of the most fundamental regulatory pathways: RNA interference. We discovered that, outside SC5314 and another genome-sequenced isolate, *C. albicans* has an active RNAi pathway that represses gene expression.

### The Limitations of Reference Strains for Understanding *C. albicans* Biology.

The use of reference strains has been instrumental in advancing our understanding of microorganisms and their roles in health, disease, and the environment, providing common baselines for researchers across the world. However, over-reliance on a limited subset of strains can introduce biases, preventing a comprehensive understanding of the biology and diversity of important microorganisms. In *C. albicans*, the wild-type reference strain SC5314 and its derivative strains are by far the most studied clinical isolates worldwide. However, SC5314 was randomly chosen as a reference strain and may not accurately represent the full spectrum of *C. albicans* biology. Indeed, extensive diversity exists between *C. albicans* natural isolates, encompassing both phylogenomic and phenotypic features (55, 56). It is emerging that the functional variability of *C. albicans* natural isolates impacts their ability to adapt to various host-niche environments, modulating the balance between commensalism and pathogenicity (32, 57, 58). It will be important to investigate the role of RNAi in regulating *C. albicans* biology, host colonization and pathogenicity in different isolates.

### Origin of the Ago1-K361 RNAi Inactive Variant.

Our data indicate that the RNAi-defective Ago-K361 variant is shared by only nine strains (seven heterozygous and two homozygous, including SC5314) of 296 analyzed. While one heterozygous strain (CEC4500) is positioned in a distinct branch of the phylogenetic tree, eight strains (comprising six heterozygous and two homozygous strains) cluster together within the same branch of the phylogenetic tree. We hypothesize that within this latter group, the homozygous RNAi-inactivating mutation arose through a two-step process. First, a heterozygous mutation occurred in the ancestor of a small group of evolutionarily related strains (Fig. 2*E*). This first event was followed by loss of heterozygosity events resulting in homozygous mutations. Homozygous and heterozygous strains with RNAi-inactivating mutations were isolated in various decades and diverse geographical locations, making it difficult to trace their ancestral origins (45, 59, 60).

### The Role of RNAi in *C. albicans*.

Subtelomeres are telomere-proximal repeat-rich regions that contain gene families and transposon-derived fragments (61, 62). While generally nonessential, subtelomeric gene families reflect organisms’ lifestyles, ensuring rapid adaptation under stress conditions. Subtelomeric gene families are involved in carbohydrate utilization in *S. cerevisiae*, and virulence in parasitic eukaryotes such as trypanosomes (61, 63, 64). *C. albicans* subtelomeres contain transposon-derived fragments and genes of the *TLO* family which encode Med2 subunits of the Mediator transcriptional regulator complex (40, 65). We found that the increased *TLO* gene expression observed in *ago1*Δ*/*Δ does not impact *C. albicans* fitness under standard laboratory growth conditions (*SI Appendix*, Fig. S2). Furthermore, in these conditions, *TLO* overexpression has a moderate impact on overall gene expression, with ~150 genes co-regulated in *dcr^RNAi^* and *ago1*Δ*/*Δ mutant strains compared to WT. We hypothesize that RNAi-mediated silencing fine-tunes *TLO* gene expression, thereby modulating *C. albicans* adaptation to changing environments. Understanding the function of individual *TLO* genes remains challenging due to technical difficulties associated with studying a large gene family with high sequence identity. Nevertheless, Tlo proteins appear to play critical roles in driving rapid, reversible adaptation to different host-niche environments by modulating transcriptional responses. Current models propose that *C. albicans* contains multiple distinct Mediator complexes, each containing functionally distinct Tlo proteins that contribute to transcriptional plasticity. Indeed, in addition to Mediator-associated Tlo proteins, SC5314 contains a substantial population of free Tlo proteins (66) which may provide a reservoir to enable fine-tuning of transcriptional responses and rapid adaptation to environmental change. *TLO* gene expression is regulated by multiple mechanisms, some of which may be redundant. Indeed, we and others have shown in SC5314 that the histone deacetylase Sir2 down-regulates *TLO* gene expression by forming hypoacetylated heterochromatin (67–69). Sir2 also promotes genome stability at subtelomeric regions (70).

This regulatory mechanism could potentially change *TLO* copy numbers in the host, altering the stoichiometry of *TLO* proteins and the composition and transcriptional activity of the Mediator complex. In the future, it will be important to investigate whether RNAi-mediated TLO silencing impacts chromatin structure.

It is likely that RNAi plays additional as-yet unexplored roles in *C. albicans* biology. As RNAi is initiated by dsRNA molecules, changes in the transcriptional landscape caused by exposure to different host niches could dramatically change RNAi targets in an environment-specific manner (71). Furthermore, it is well-documented that RNAi plays important roles in regulating genome stability by controlling transposon activity, heterochromatin formation, and triggering drug resistance by seeding reversible epimutations (71–76). *C. albicans* lacks the canonical heterochromatin structure that is associated with RNAi activity (77). In this study, we identified sRNA clusters at some transposable elements including full-length and truncated retrotransposons and DNA transposons. Future studies will unveil the role of *C. albicans* RNAi at transposable elements and will establish whether this activity controls genome plasticity.

RNAi can also act as an antiviral defense mechanism by degrading RNA viruses (18). RNA viruses have not been described in *C. albicans,* and it remains to be established whether *C. albicans* RNAi has an antiviral defense role.

Given that an active RNAi machinery is predicted to be present in other pathogenic *Candida* species (*SI Appendix*, Table S3), our study significantly contribute to on our understanding of a clinically important but poorly understood group of pathogens.

## Materials and Methods

### Media and Culture.

Strains used in this study are listed in *SI Appendix*, Table S1. All strains were grown in YPD containing 1% Yeast Extract, 2% Peptone, 2% Dextrose, 0.1 mg/mL adenine and 0.08 mg/mL uridine. Routine culture was performed at 30 °C, 180 rpm shaking. Growth curves were performed in YPD at 30 °C or 37 °C in a Spectro Star Nano, absorbance plate reader (BGM Labtech). Analysis was made using three biological replicates and graphs were generated using Microsoft Excel.

### *C. albicans* CRISPR-Cas9 Engineered Strains.

Oligos used for CRISPR-Cas9 genome editing are listed in *SI Appendix*, Table S2 and indicated in brackets in the following text.

Mutants were created using the CRISPR-Cas9 system HIS1-FLP and the AddTag approach as previously described (78, 79). Briefly, to delete *AGO1*, we designed a guide to a sequence, gRNA-PAM1 (AB1146), to induce a double-stranded cut by Cas9 in the middle of the *AGO1* gene. A repair template with a sequence homology of 76 to 86 bp around the *AGO1* gene was used to delete the whole ORF (AB1173 + AB1174 for GC75, P37039, and P75016 isolates or AB1154 + AB1154 for SC5314). For all isolates, except SC5314, the repair template included the specific sequence “ADDTAG1” to introduce a target sequence with a new PAM. To reintroduce *AGO1* gene, the new ADDTAG1 target sequence (AB1178) was used to induce a double-stranded cut by Cas9. In SC5314, a target sequence spanning from the upstream and downstream region of *AGO1* (AB1391) was used to induce the double-stranded cut. For all, a repair template of the full *AGO1* gene including 220 bp before and 468 bp after the gene was designed by PCR (AB1151 + AB1311) on genomic DNA from either SC5314 (Ago1-K361) or GC75 (Ago1-E361). This technique allowed us to change the original *AGO1* allele from one isolate to another.

To generate *dcr1^RNA^*^i^ mutant strain, the target sequence, gRNA-PAM3 (AB1309), induced a double-stranded cut by Cas9 six nucleotides upstream to the new stop codon after Pro512 inserted by the repair template (AB1300 + AB1301), resulting in a shorter Dcr1 protein without the last dRBD2 (*SI Appendix*, Fig. S7*A*).

*C. albicans* was chemically transformed using an optimized Lithium-Acetate method (80) with 2 µg of plasmid pADH99 (the first part of the cassette to insert into *C. albicans* genome and containing the gene coding for Cas9) digested by MssI, 50 µL of fragment C (the second part of the cassette to insert into *C. albicans* genome), 100 µg of salmon sperm DNA (Sigma D7656), and a minimum of 1 µg of Repair Template. Transformants were selected on 200 µg/mL Nourseothricin and after PCR confirmation, the mutants were passaged in YP-Maltose to induce the removal of the Cas9 cassette.

Transformants were screened using oligo pairs AB992 + AB991 or AB1177 + AB1156 and confirmed by Sanger Sequencing (Eurofins) using oligos AB992.

### RNA Extraction.

RNA was extracted from cells growing in exponential phase in YPD at 30 °C. Small RNA was extracted using mirVana miRNA isolation kit (ThermoFisher) following the protocol for organic extraction and was then treated with DNAse I (ThermoFisher). Total RNA was extracted using two different kits: either the MasterPure Yeast RNA Purification kit (Biosearch Technologies) or the EZNA Yeast RNA Kit (Omega Bio-Tek) following the protocol with DNAse treatment. All RNA samples were controlled on TapeStation (BioAgilent).

### RT-qPCR Analysis.

cDNA was made with 100 ng of RNA using UltraScript Reverse Transcriptase (PCR Biosystems). Quantitative PCR (qPCR) was performed using qPCR SyGreen Mix (PCR Biosystems) with 1 µL of cDNA using BIO-RAD CFX Connect instrument. AB354 and AB354 are Pan-*TLO* primers and *ACT1* primers AB174 and AB176 are used as housekeeping gene. The Relative Expression Analysis Tool (qRAT) was used to analyze and generate graphs using the data from at least three biological replicates (81–83).

### Bioinformatics Analysis.

#### Structural prediction.

Structural prediction of *C. albicans* Ago1 was performed using AlphaFold.ipynb–Colaboratory (google.com) and visualized with ChimeraX. Protein alignments were performed using Muscle with default parameters and visualized using Jalview. The following proteins were used for the protein alignments: *C. albicans* (CANAL) Ago1p (UniProt: A0A1D8PMK0); *Naumovozyma castellii* (NAUCA) Ago1 (UniProt: D5SHI8); *Schizosaccharomyces pombe* (SCHPO) Ago1 (UniProt: O74957); *Vanderwaltozyma polyspora* (VANPO) Ago1 (UniProt: A7TMA9); *Caenorhabditis elegans* (CAEEL) RDE-1 (UniProt: G5EEH0); *Drosophila melanogaster* (DROME) Ago1 (UniProt: Q27IR0) and Ago2 (UniProt: Q9VUQ5); and *Homo sapiens* (HUMAN) Ago2 (UniProt: Q9UKV8)

#### RNA-seq and sRNA-seq analysis.

RNA-seq and sRNA-Seq analyses were performed in at least three biological replicates. Raw reads were processed to verify their quality scores and to confirm the absence of adaptor sequences using FastQC v0.12.1 (https://www.bioinformatics.babraham.ac.uk/projects/fastqc/). Long RNA reads were mapped using HISAT2 version 2.2.1 (84) and multimapping reads were only mapped once to the reference genome adjusting the parameter **-*k* to *1*. Small-RNA reads were mapped using Bowtie2 version 2.2.5 (85). For each sample, genome-guided transcriptome assembly was performed using StringTie v2.2.1 (86). The reference genome and annotation for *C. albicans* SC5314 (assembly 22) were obtained from Candida Genome Database (CGD, last accessed on June 2023) (87), and considering that the genome sequence of *C. albicans* is phased, only haplotype A was used for the read mapping. For the annotation of noncoding and repetitive elements, the data were obtained from a file including “other features genomic” in the CGD (last accessed on November 2023) and was modified in order to extract the IDs of every element included in the haplotype A and their genomic positions.

To assess the expression levels of both long- and small-RNA and compare them to those of protein-coding, read counts were estimated and normalized using StringTie v2.2.1 with the parameters **-*eBG.* Only the abundance of given reference transcripts was estimated, and these counts were normalized considering gene length, resulting in fragments per kilobase per million mapped reads (FPKM). Differential gene expression analysis (DGEA) was performed using two R (version 4.2.2) packages: tximport (88) and DESeq2 (89). First, tximport was used to import and summarize transcript-level abundance estimates by reversing the formula that StringTie used to calculate coverage. Second, a prefilter was applied to remove low count genes, removing all genes with less than 10 reads mapping them. Finally, the matrix with the estimated and filtered counts was used as input for DGEA. The standard DGEA, which is wrapped into a single function of the package DESeq2, was performed to extract a results table with log2 fold changes, *P*-values and q-value (Dataset S1), which were used to generate heatmaps using R studio. Similarly, to assess the expression levels of both long- and small-RNA and compare them to those of noncoding and repetitive elements, the coordinates of all reads mapped to the reference genome were intersected to those of the noncoding and repetitive elements using BEDtools (90). After this, the read counts were organized to form the matrix needed to perform DGEA, starting from the prefilter step to remove low-count genes.

For the visualization of small-RNA lengths in specific regions of the *C. albicans* reference genome, sRNA_Viewer (https://github.com/MikeAxtell/sRNA_Viewer) was used with the following parameters: *-c -b -p.* This software includes both, the normalization (reads per million—RPM) of each mapping track and the generation of the plots. In order to adjust the Y-axis of the figures, which is scaled by default, the original script was modified and the following parameter was added after the line 324: *coord_cartesian(ylim = c(Ymin, Ymax))*, where *Ymin* and *Ymax* are the minimum and maximum values of the Y-axis, respectively.

The mapped reads to the reference genome were filtered depending on their length into two groups: reads between 18 and 30 nucleotides (nt) and reads between 20 and 23 nt. All reads outside these ranges were removed. For the 18 to 30 nt group, the 5′ nucleotide of all reads was manually inspected and the coverage of each type of 5′- nt against the total number of reads was calculated for each read length. Additionally, a *t* test was performed to verify how significant were the differences between WT and DCR read length distributions. This *t* test was performed twice, for both ranges of sizes, 18 to 30 nt and 20 to 23 nt.

#### Quantification of siRNA peaks.

The *C. albicans* genome was divided in windows of 500 bp to localize those regions in the genome containing sRNA peaks. For this purpose, reads were filtered to only allow those whose length was 22 and/or 23 nucleotides to map to the reference genome. Next, the coordinates of the filtered reads were intersected with the 500 bp windows to count the number of reads per window. Finally, the read counts per window were intersected with the coordinates of both, protein-coding and noncoding and repetitive elements.

#### Phylogenetic analysis.

Single nucleotide polymorphisms in the *AGO1* gene of 296 genome-sequenced *C. albicans* isolates including SC5314 (45, 91) were retrieved, and the impact on the Ago1 protein sequence was evaluated in each of the 296 isolates. A phylogenetic tree was constructed using RAxML (92) and data for 215794 high-confidence SNPs available for all 296 strains. All sequences of *AGO1*, *DCR1,* and *CDL1* of *Candida tropicalis*, *Candida parapsilosis* and *Candida dubliniensis* were retrieved from NCBI Genomes database and compared using MUSCLE Mutliple sequence alignment tool (93).

## Supplementary Material

Appendix 01 (PDF)

Dataset S01 (XLSX)

Dataset S02 (XLSX)

Dataset S03 (XLSX)

Dataset S04 (XLSX)

## Data Availability

RNA-seq and sRNA-seq data have been deposited in NCBI (https://dataview.ncbi.nlm.nih.gov/object/PRJNA1074533?reviewer=k150hn9dsd47h9lu3j2v4ihpep) (94).
