# Supplementary material for: Identification of an active RNAi pathway in Candida albicans
Source: Proc Natl Acad Sci U S A. 2024 Apr 16;121(17):e2315926121. doi: 10.1073/pnas.2315926121 (PMC11047096; doi:10.1073/pnas.2315926121)
Supplement: Supplementary file 1 — Appendix 01 (PDF) [file pnas.2315926121.sapp.pdf]

PNAS

Discovery of an active RNAi pathway in *Candida albicans*

Elise Iracane[1], Cristina Arias-Sardá[1], Corinne Maufrais[2], Iuliana V. Ene[3], Christophe d'Enfert[4] and Alessia Buscaino[1 *]

[1] Kent Fungal Group, School of Biosciences, Division of Natural Sciences
University of Kent Canterbury, Kent CT2 7NZ

[2] Institut Pasteur, Université Paris Cité, Bioinformatic Hub, Paris, France

[3] Institut Pasteur, Université Paris Cité, Fungal Heterogeneity Group, Paris, France

[4] Institut Pasteur, Université Paris Cité, INRAE USC2019, Fungal Biology and Pathogenicity Unit, Paris, France

*Corresponding author

Email: A.Buscaino@kent.ac.uk

This PDF file includes:

Figures S1 to S10
Tables S1 to S3
Legends for Datasets S1 to S4
SI References

Other supporting materials for this manuscript include the following:

Datasets S1 to S4

Supplementary Figure S1 *Iracane et al*

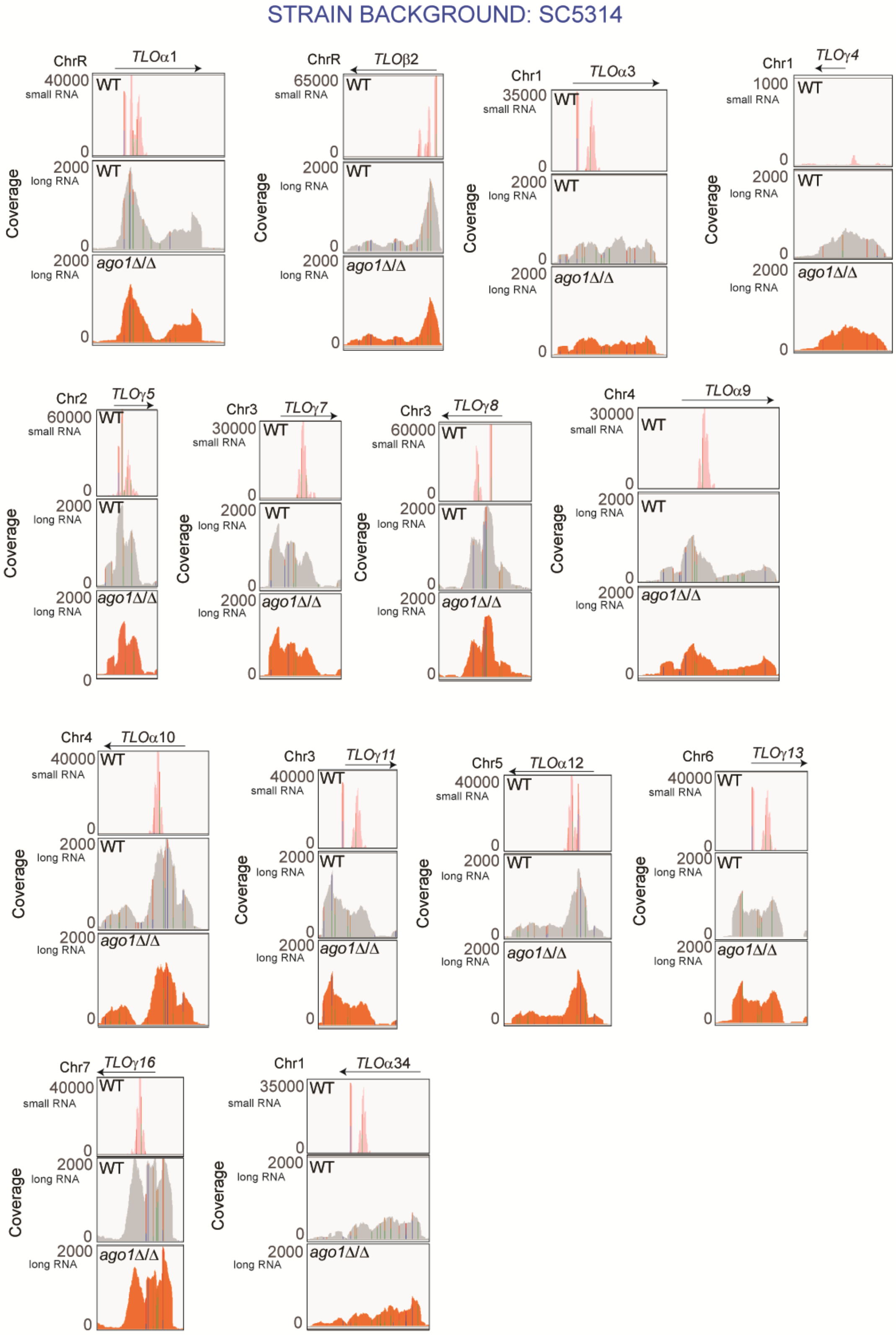

Fig. S1. Strain background: SC5314. *Top:* Small RNA coverage at *TLO* genes in the SC5314 WT strain*; Middle and Bottom:* Long RNA coverage at *TLO* genes in the WT and *ago1Δ/Δ* strains.

Supplementary Figure S2

STRAIN BACKGROUND: SC5314

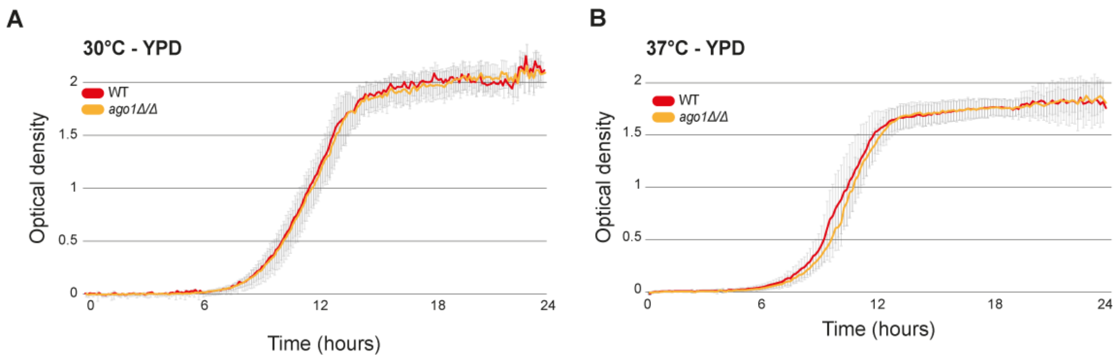

Fig. S2. (A) Growth curve of SC5314 wild type and *ago1Δ/Δ* at 30°C. **(B)** Growth of SC5314 wild type and *ago1Δ/Δ* at 37°C. All samples were done in biological triplicates with technical triplicates for each. Error bars: standard deviation

Supplementary Figure 3 *Iracane et al*

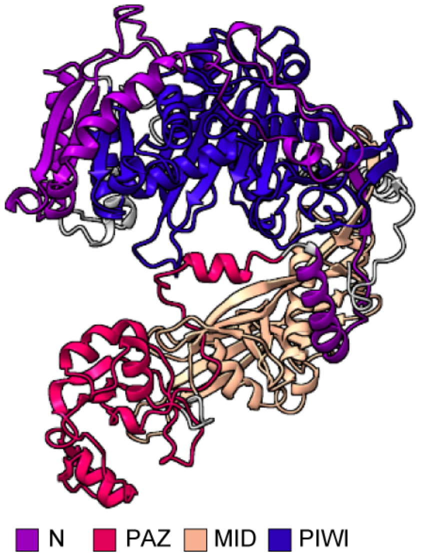

Fig. S3 Alpha Fold representation of *C. albicans* Ago1, colour coded by domain (N-terminal in purple, PAZ domain in red, MID domain in beige and PIWI domain in blue).

Supplementary Figure 4 **MID** *Iracane et al*

A

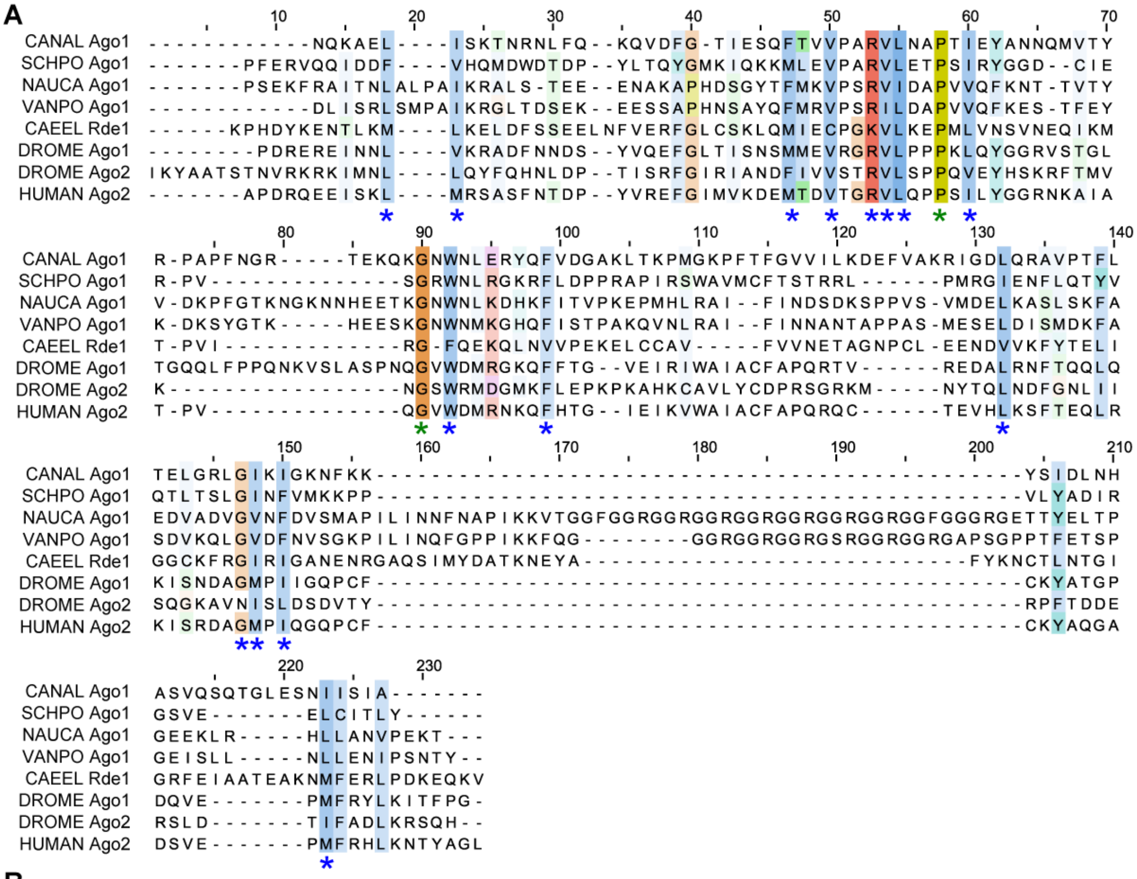

B

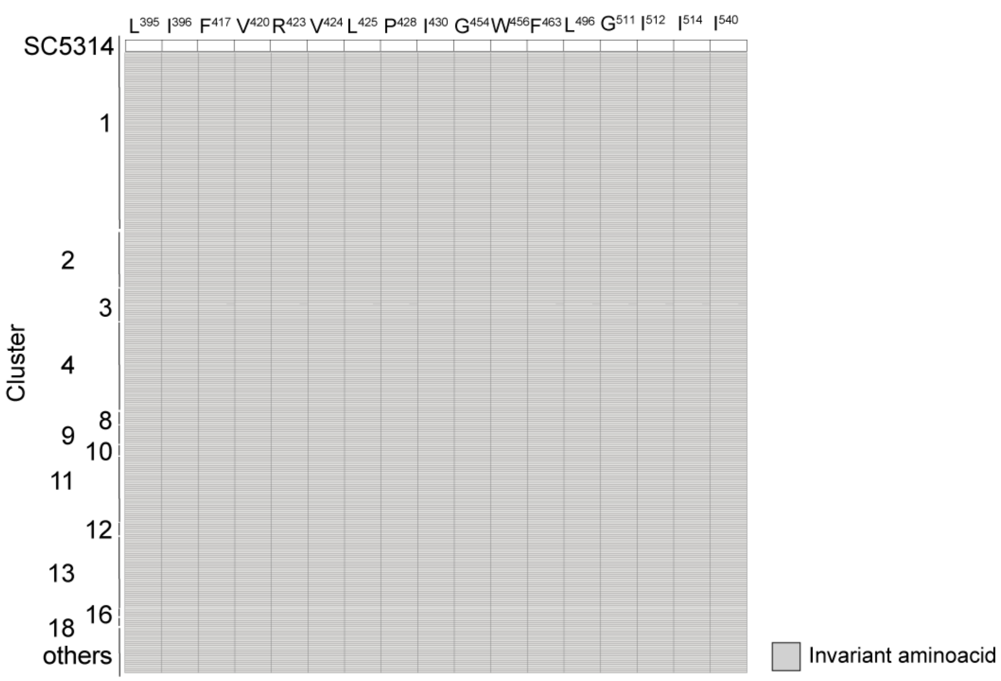

Fig S4. (**A**) Alignment of MID domain from 8 eukaryotic species. CANAL: *C. albicans* Ago1 aa 390 - 544; SCHPO: *Schizosaccharomyces pombe* Ago1 aa 350-487; NAUCA: *Naumovozyma castellii* Ago1 aa 726-936; VANPO: *Vanderwaltozyma polyspora* Ago1 aa 694-888; CAEEL: *Caenorhabditis elegans* Rde-1 aa 444-618; DROME: *Drosophila melanogaster* Ago1 aa 482-638 and Ago2 737-886; HUMAN: *Homo sapiens* Ago2 aa 372-515. Alignment visualised on Jalview, residues coloured by functional conservation. Blue stars: conserved residues; green stars: identical residues (**B**) Residues spanning from 395 to 540 of Ago1 MID domain from 295 *C. albicans* isolates compared to SC5314 reference sequence (top of the graph). The isolates are sorted by clusters. In grey: invariant residues; in blue: heterozygous variant; in purple: homozygous variant.

Supplementary Figure 5

Iracane et al

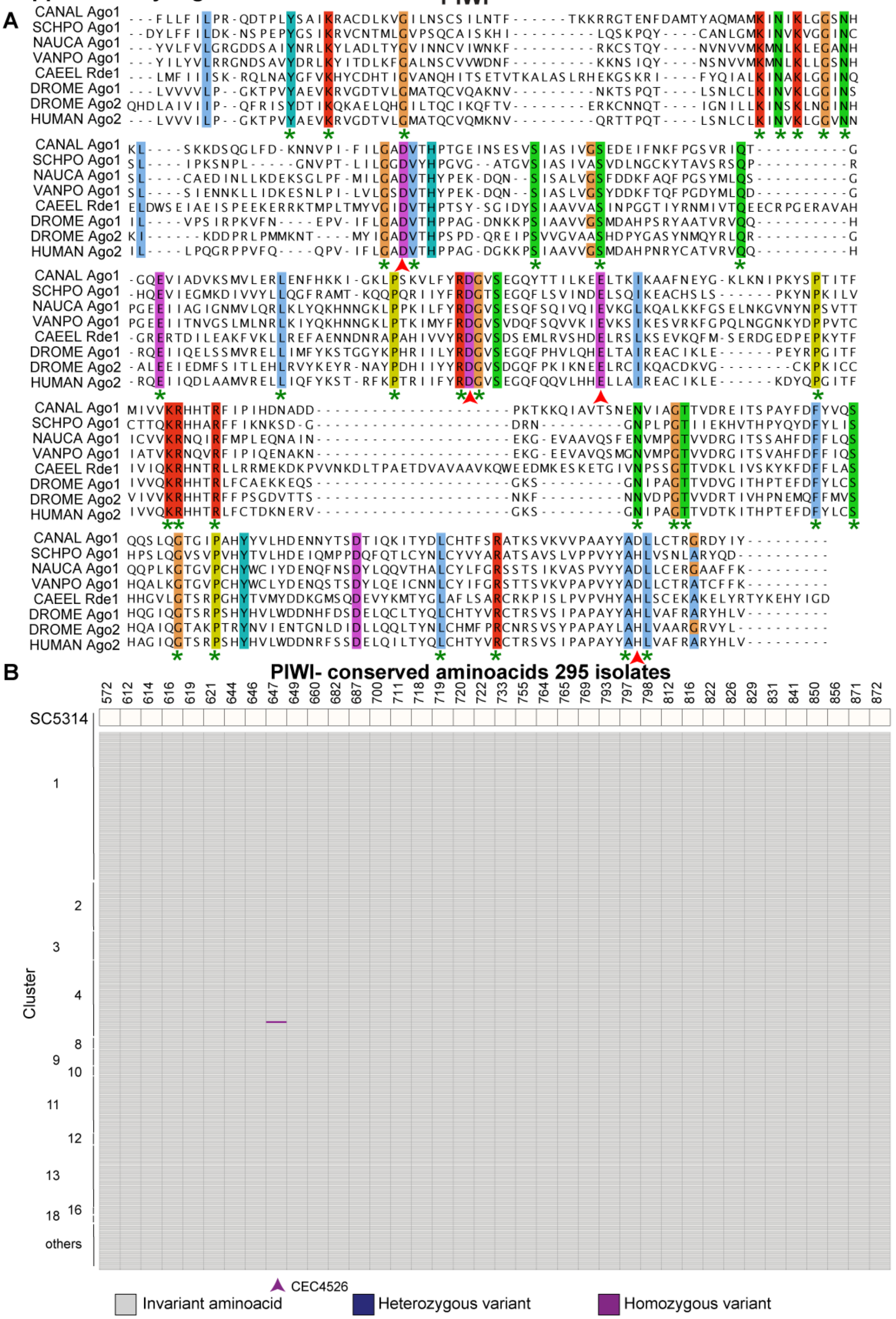

Fig S5. (**A**) Alignment of PIWI domain from 8 eukaryotic species. CANAL: *C. albicans* Ago1 aa 555-882; SCHPO: *Schizosaccharomyces pombe* Ago1 aa 499-798; NAUCA: *Naumovozyma castellii* Ago1 aa 937-1258; VANPO: *Vanderwaltozyma polyspora* Ago1 aa 888-1209; CAEEL: *Caenorhabditis elegans* Rde-1 aa 619-994; DROME: *Drosophila melanogaster* Ago1 aa 641-943 and Ago2 885-1186; HUMAN: *Homo sapiens* Ago2 aa 517-818. Alignment visualised on Jalview, residues coloured by functional conservation. Green stars: identical residues; red arrows: catalytic tetrad. (**B**) Main residues spanning from 572 to 872 of Ago1 PIWI domain from 295 *C. albicans* isolates compared to SC5314 reference sequence (top of the graph). The isolates are sorted by clusters. In grey: invariant residues; in blue: heterozygous variant; in purple: homozygous variant.

Supplementary Figure 6

Iracane et al.

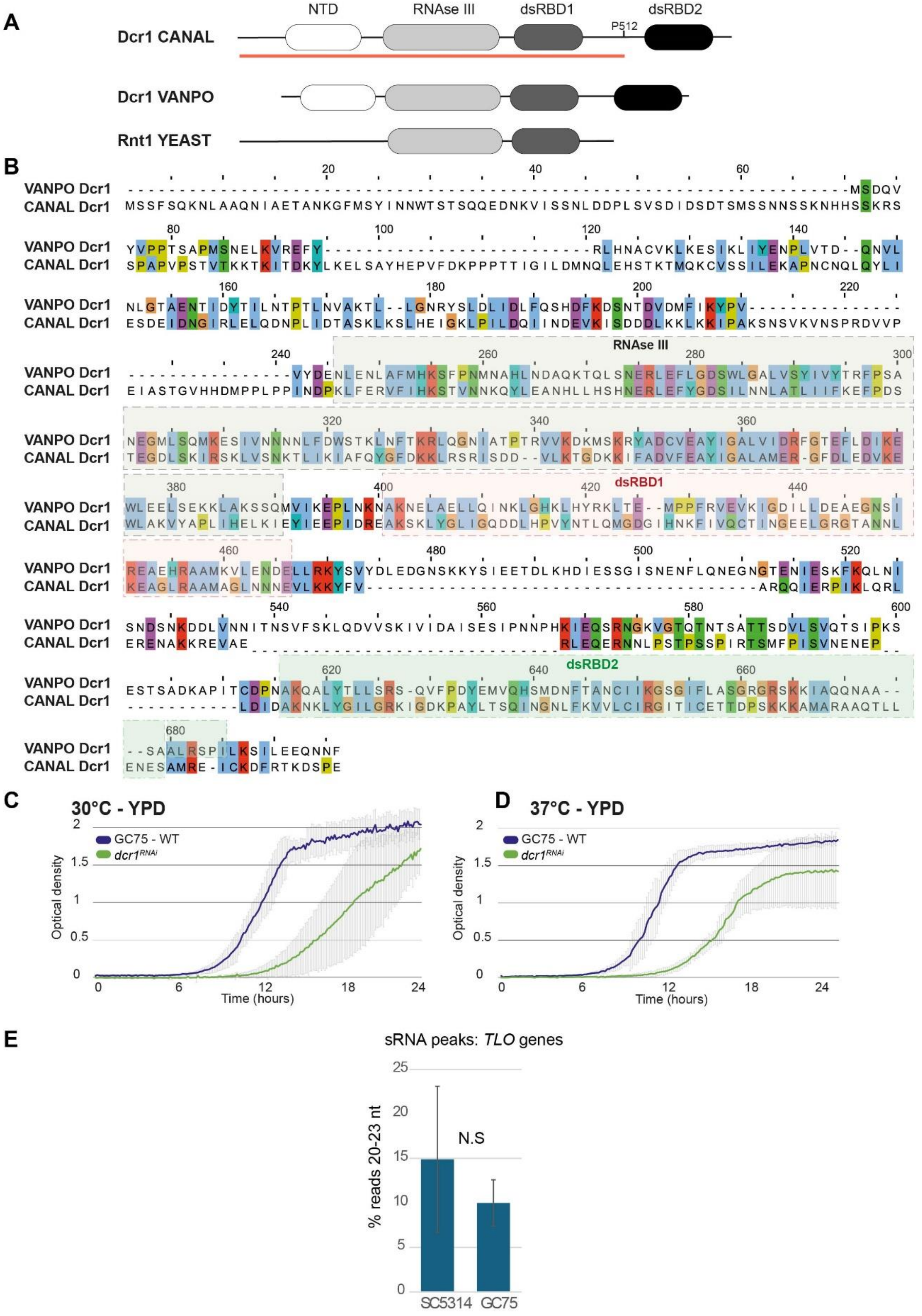

Fig S6. (A) Graphical representation of *C. albicans* Dcr1 (Dcr1 CANAL), *V. polyspora* Dcr1 (Dcr1 VANPO) and the canonical Rnt1 protein in *S. cerevisiae* (Rnt1 YEAST); orange line: protein encoded by the *dcr1*^{RNAi} mutant strain. **(B)** Alignment of Dicer proteins from *Vanderwaltozyma polyspora* (VANPO Dcr1) and *C. albicans* (CANAL Dcr1). Conserved domains are highlighted in grey (RNAse III), red (dsRBD1) or green (dsRBD2). **(C)** and **(D)** Growth of GC75 wild type and *dcr1*^{RNAi} at 30°C and 37°C, respectively, over 24h. All samples were done in biological triplicates with technical triplicates each. Error bars: standard deviation. **(E)** 20 to 23 nt sRNA peaks at *TLO* genes in SC5314 and GC75 isolates, expressed in percentage of total number of reads of 20-23 nt. N.S: not selective.

Supplementary Figure 7

Iracane et al.

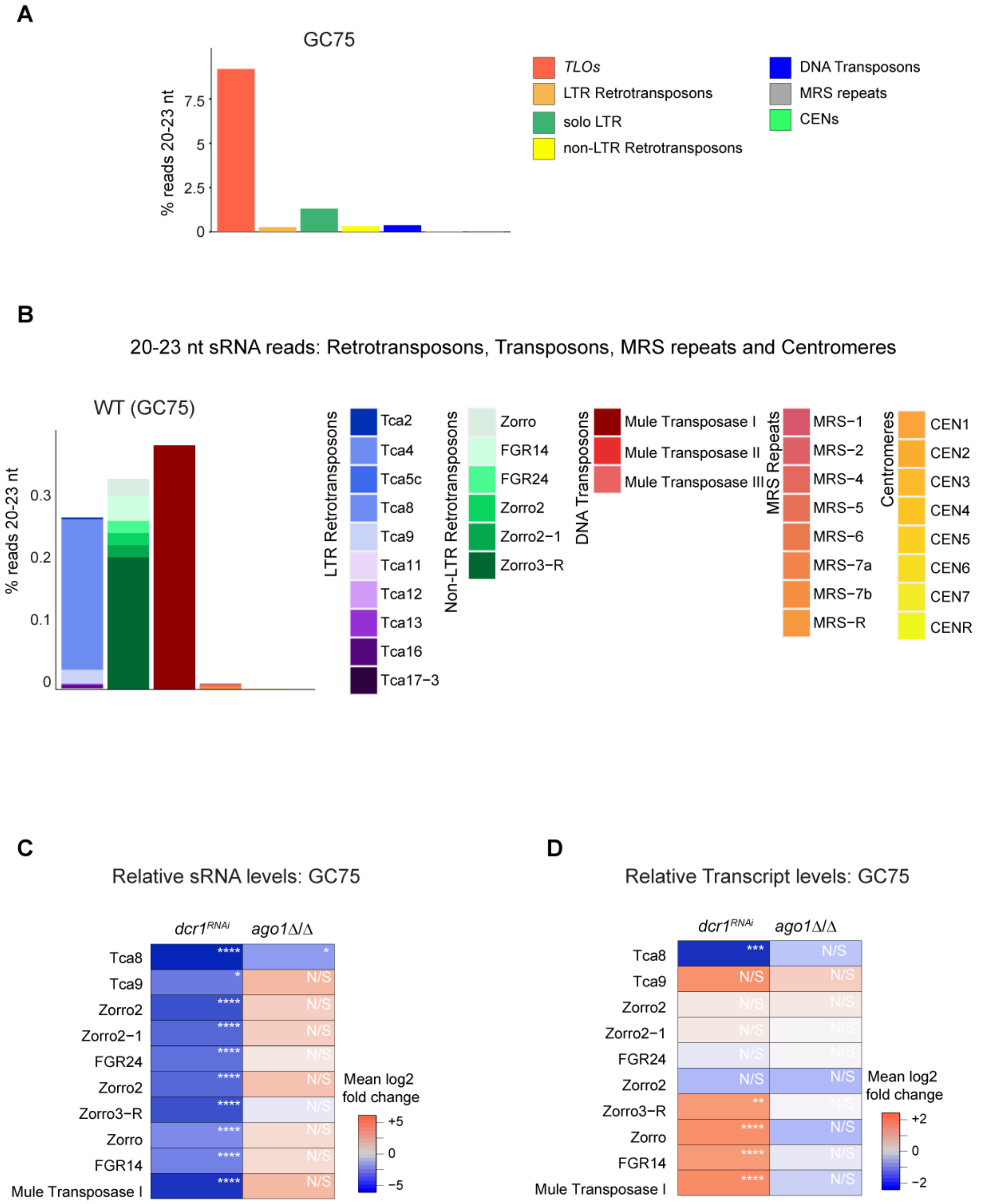

Fig. S7. (A) Percentage of 20 to 23 nt reads mapping to TLO genes, LTR-retrotransposons, solo LTRs, non-LTR retrotransposons, DNA transposons, MRS repeats and centromeres (CEN) in GC75 wild type **(B)** Percentage of 20 to 23 nt reads mapping to LTR-retrotransposons, non-LTR retrotransposons, DNA transposons, MRS

repeats and centromeres (CEN) in GC75 wild type; all peaks are expressed in percentage of 20-23 nt reads and color-coded per categories **(C)** Heat map of sRNA log2 fold change in *dcr1*^{RNAi} (GC75) and *ago1*Δ/Δ (GC75) for selected retrotransposons and transposons from sRNA-seq data. n.s.= not significant ; $p < 0.05$ **(D)** Relative transcript levels of selected retrotransposons and transposons in *dcr1*^{RNAi} (GC75) and *ago1Δ/Δ* (GC75). n.s.= not significant; $p < 0.05$: *; $p < 0.005$: **; $p < 0.0005$: ***; $p < 0.00001$: ****.

Supplementary Figure 8 *Iracane et al*

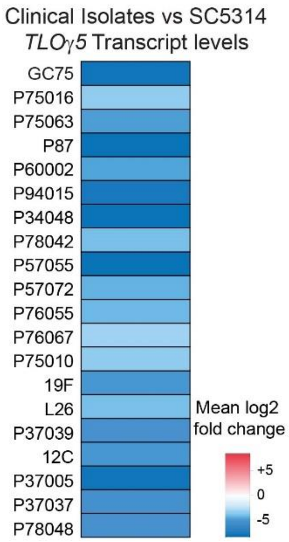

Fig. S8. Relative transcript level of *TLOγ5* in 20 *C. albicans* isolates compared to SC5314. RNA-seq data from Wang et. al (1).

Supplementary Figure 9 *Iracane et al*

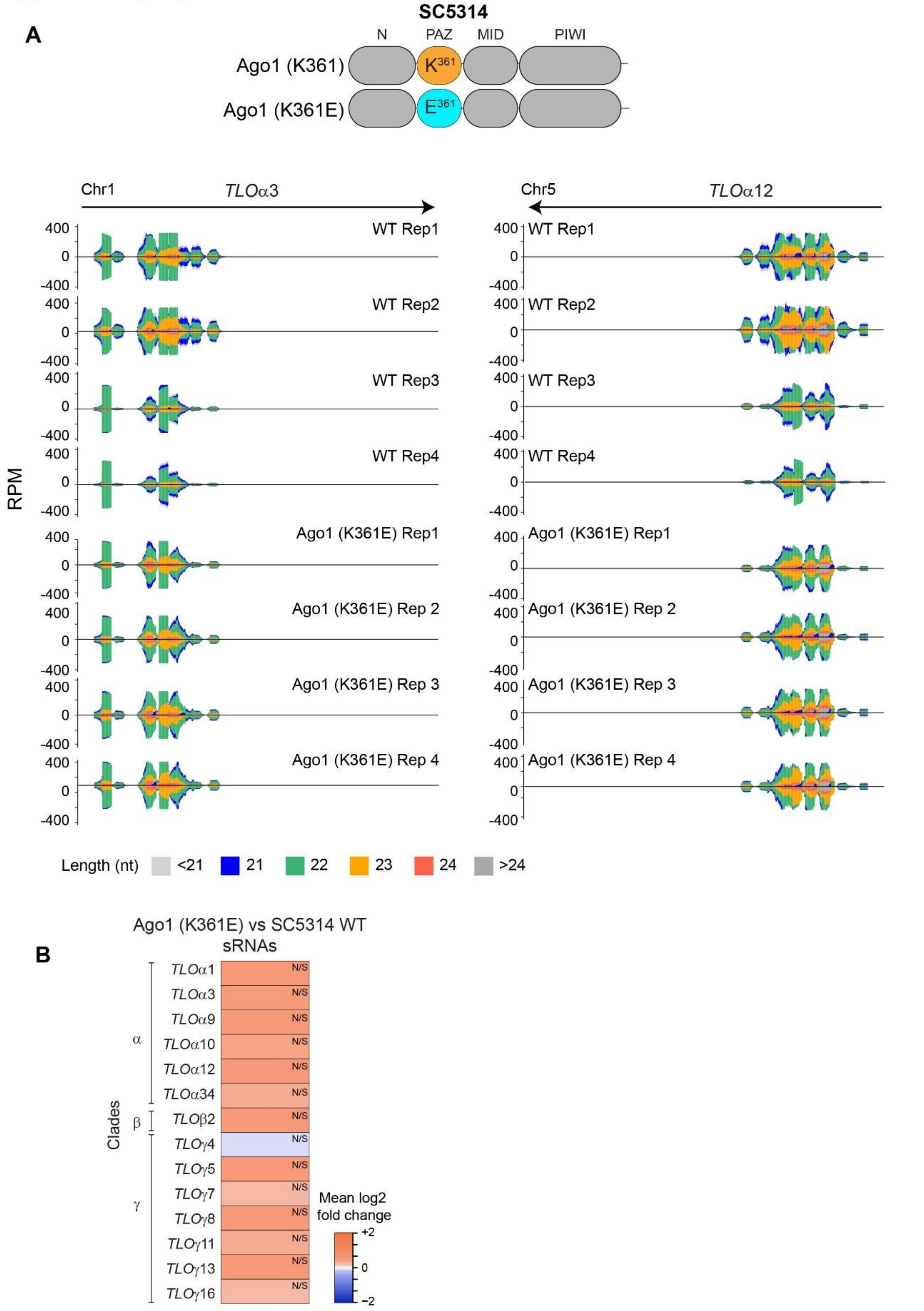

Fig. S9. Small RNA levels in SC5314 Ago1-K361E at TLOs loci. **(A)** Graphical representation of Ago1 variants in SC5314. **(B)** Small RNA profiling of loci *TLOα3* and

TLOα12 in four biological replicates (Rep1, Rep2, Rep3 and Rep4) of WT (SC5314) and Ago1-K361E strains. **(C)** Heat map of sRNA log2 fold change in SC5314 Ago1-K361E for *TLO* loci from sRNA-seq data; n.s.: not significant.

Supplementary Figure 10

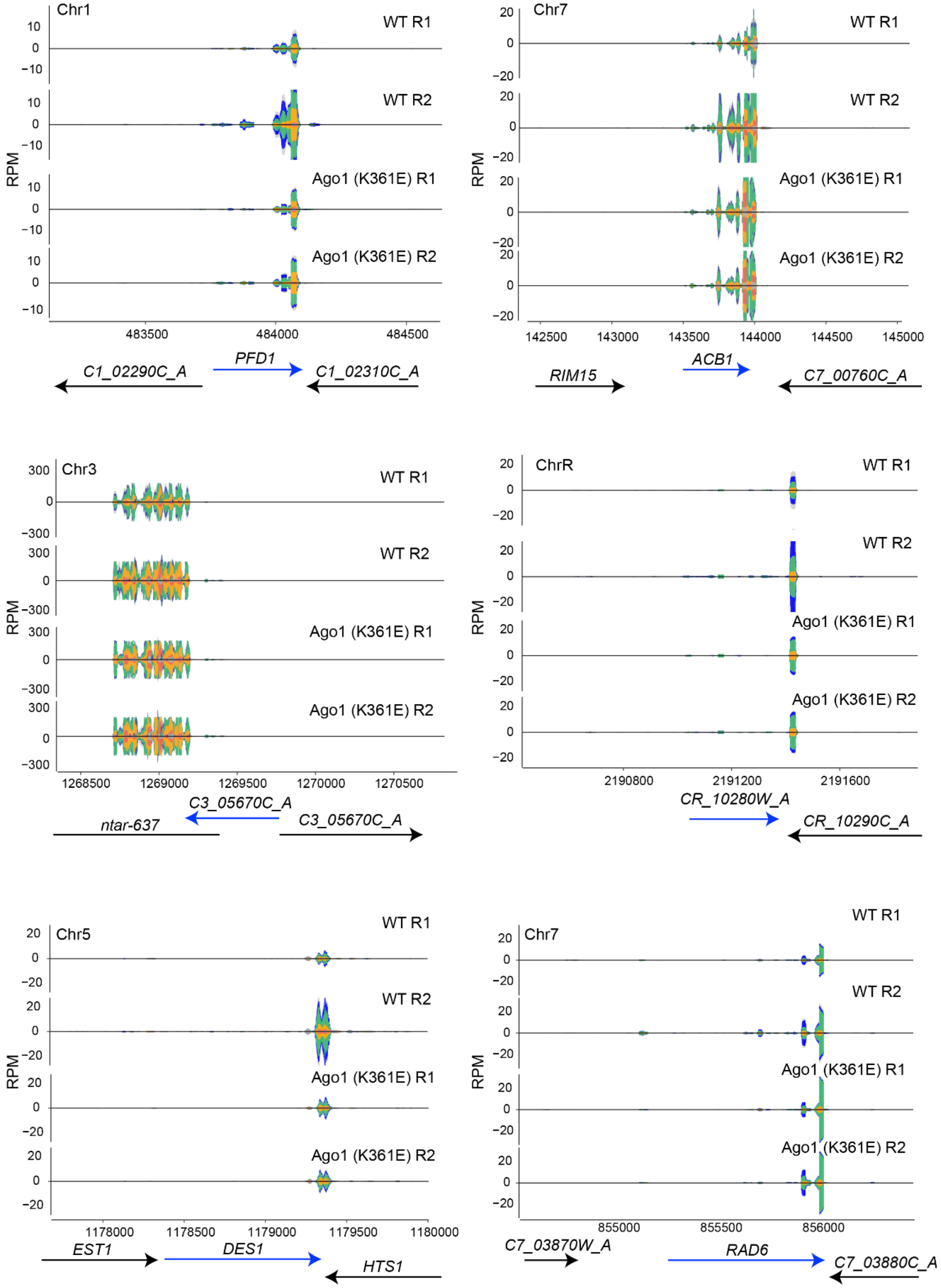

Fig. S10. Small RNA levels in SC5314 Ago1-K361E at selected loci. Small RNA profiling of loci *PFD1, ACB1, C3_05670C_A, CR_10280W_A, DES1* and *RAD6* in two biological replicates (R1 and R2) of WT (SC5314) and Ago1-K361E strains.

Strain Number	Strain name	Parental strain	Genotype	Reference
AB55	SC5314		Wild Type	(2)
AB846	GC75		Wild Type	(3)
AB833	P37039		Wild Type	(4)
AB832	P75016		Wild Type	(3)
AB853	*ago1 Δ/Δ*	SC5314	*ago1Δ/ago1Δ*	This study
AB1119	Ago1 - K361E	SC5314	*ago1Δ::AGO1-E361/ago1Δ::AGO1-E361*	This study
AB895	*ago1 Δ/Δ*	GC75	*ago1Δ::ADDTAG1/ago1Δ::ADDTAG1*	This study
AB1122	Ago1 - E361K	GC75	*ago1Δ::ADDTAG1::AGO1-K361/ago1Δ::ADDTAG1::AGO1-K361*	This study
AB1123	Ago1 - Reconstituted strain	GC75	*ago1Δ::ADDTAG1::AGO1-E361/ago1Δ::ADDTAG1::AGO1-E361*	This study
AB1240	*ago1 Δ/Δ*	P37039	*ago1Δ::ADDTAG1/ago1Δ::ADDTAG1*	This study
AB940	*ago1 Δ/Δ*	P75016	*ago1Δ::ADDTAG1/ago1Δ::ADDTAG1*	This study
AB1118	*dcr1*^{RNAi}	GC75	*dcr1ΔdsRBD2 / dcr1ΔdsRBD2*	This study

Supplementary Table 1: Strains used in the study.

Oligonucleotide name	Sequence
AB174	CTACGTTTCCATTCAAGCTGTT
AB176	AAACTGTAACCACGTTCAGACA
AB354	CTAGCCCCAACAACGAACTA
AB355	GGCCTCTTCTTCACGTTTCT
AB991	ATCACCAATACGTTTAGCAACA
AB992	TTGCGGTTATGGGATCCCAA
AB1146	CGTAAACTATTTTTAATTTGTCCTCATCAACAATATAAGGGTT TTAGAGCTAGAAATAGC
AB1151	ATCCTCCCTGTCCTCTCCTT
AB1154	TATAGTTGTATCAATCCTCCGACCTTCGACCTCGCCCCGTGT CCCACTCCCCTACCACGTCTCTGGCTATTTAACAATCTATTA TTTGGTGATAGT
AB1155	TTAATAAAAAAAAAAAAACGAAAAAAAAAAAACGAAGGAGATG ATGAAGAAGAAGAAATGAATAAAAACAAAAACTAACTATCAC CAAATAATAGAT
AB1156	ACGAAGGAGATGATGAAGAAGA
AB1173	TCAATCCTCCGACCTTCGACCTCGCCCCGTGTCCCACTCCC CTACCACGTCTCTGGCTATTTAACAATCTATTATTCGTACGC TGCAGGTCGACAG
AB1174	AAAAACGAAAAAAAAAAACGAAGGAGATGATGAAGAAGAAG AAATGAATAAAAACAAAAACTAACTATCACCACCACTGTCGA CCTGCAGCGTACG
AB1177	CTGCAGGTCGACAGTGGTT
AB1178	CGTAAACTATTTTTAATTTGCGTACGCTGCAGGTCGACAGGT TTTAGAGCTAGAAATAGC
AB1300	AGAGGGAGGTCGCAGAAAGATTAGAACAAGAAAGGAATAAC CTTCCTTCTACACCGCTGTCGCCATAGATAGATAGCGTACG CTGCAGGTCGACA
AB1301	GTAAAATTTAATGCATGTTTAGGGAATAACTATACATGAATAC GTCTATAATGTAAATAAACAAATAATACCACTGTCGACCTGC AGCGTACGCT
AB1311	ACCCCTATTGAAAACCCCAA
AB1391	CGTAAACTATTTTTAATTTGCTATTTAACAATCTATTATTGTTT TAGAGCTAGAAATAGC

Supplementary Table 2: Oligonucleotides used in the study

	Ago1		Dcr1	Cdl1
	PAZ domain	PIWI domain	Active catalytic sites	Inactive sites
	"RNAi active" E (CaE361)	Active catalytic sites		
C. tropicalis 43 strains	100%	No variability	No variability	No variability
C. parapsilosis 35 strains	100 %	No variability	No variability	No variability
C. dubliniensis 4 strains	100%	No variability	No variability	No variability

Supplementary Table 3: Ago1, Dcr1 and Cdl1 variability within *Candida tropicalis*, *Candida parapsilosis* or *Candida dubliniensis* isolates at conserved residues. Ago1 variability based on *C. albicans* Ago1 conserved residues described in Fig 2(A) and Fig S5(A) (red arrows). Dcr1 and Cdl1 variability and conserved residues in RNAse-III domain based on Bernstein et al, Fig1C (5).

Dataset S1:

RNA-seq and sRNA-seq differential gene expression

Dataset S2:

Small RNA peaks in SC5314 and GC75 isolates

Dataset S3:

Candida albicans SC5314 isolates: SNP calling for *AGO1.*

Dataset S4:

SNP calling for *AGO1* in 296 *Candida albicans* isolates.

SI References

1. J. M. Wang, *et al.*, Intraspecies Transcriptional Profiling Reveals Key Regulators of *Candida albicans* Pathogenic Traits. *mBio* **12** (2021).
2. A. M. Gillum, E. Y. H. Tsay, D. R. Kirsch, Isolation of the Candida albicans gene for orotidine-5′-phosphate decarboxylase by complementation of S. cerevisiae ura3 and E. coli pyrF mutations. *Mol Gen Genet* **198**, 179–182 (1984).
3. R. A. Hajjeh, *et al.*, Incidence of Bloodstream Infections Due to *Candida* Species and In Vitro Susceptibilities of Isolates Collected from 1998 to 2000 in a Population-Based Active Surveillance Program. *J Clin Microbiol* **42**, 1519–1527 (2004).
4. S. R. Lockhart, B. D. Reed, C. L. Pierson, D. R. Soll, “Most Frequent Scenario for Recurrent Candida Vaginitis Is Strain Maintenance with ‘“Substrain Shuffling”’: Demonstration by Sequential DNA Fingerprinting with Probes Ca3, C1, and CARE2” (1996).
5. D. A. Bernstein, *et al.*, Candida albicans Dicer (CaDcr1) is required for efficient ribosomal and spliceosomal RNA maturation. *Proc Natl Acad Sci U S A* **109**, 523–528 (2012).
